# Looking ahead: ethical and social challenges of somatic gene therapy for sickle cell disease in Africa

**DOI:** 10.1038/s41434-023-00429-7

**Published:** 2023-11-27

**Authors:** Nchangwi Syntia Munung, Obiageli E. Nnodu, Patrick Ohiani Moru, Akpaka A. Kalu, Benido Impouma, Marsha J. Treadwell, Ambroise Wonkam

**Affiliations:** 1 Division of Human Genetics, Faculty of Health Sciences, University of Cape Town, Cape Town, South Africa; 2 Centre of Excellence for Sickle Cell Disease Research and Training (CESRTA), University of Abuja, Abuja, Nigeria; 3 World Health Organization Regional Office for Africa, Brazzaville, Republic of Congo; 4 Department of Pediatrics, Division of Hematology, University of California San Francisco, Oakland, CA USA; 5 McKusick-Nathans Institute & Department of Genetic Medicine, Johns Hopkins University School of Medicine, Baltimore, MD USA

**Keywords:** Genetics, Haematological diseases

## Abstract

Somatic gene therapy will be one of the most exciting practices of genetic medicine in Africa and is primed to offer a “new life” for persons living with sickle cell disease (SCD). Recently, successful gene therapy trials for SCD in the USA have sparked a ray of hope within the SCD community in Africa. However, the high cost, estimated to exceed 1.5 million USD, continues to be a major concern for many stakeholders. While affordability is a key global health equity consideration, it is equally important to reflect on other ethical, legal and social issues (ELSIs) that may impact the responsible implementation of gene therapy for SCD in Africa. These include informed consent comprehension, risk of therapeutic misestimation and optimistic bias; priorities for SCD therapy trials; dearth of ethical and regulatory oversight for gene therapy in many African countries; identifying a favourable risk-benefit ratio; criteria for the selection of trial participants; decisional conflict in consent; standards of care; bounded justice; and genetic tourism. Given these ELSIs, we suggest that researchers, pharma, funders, global health agencies, ethics committees, science councils and SCD patient support/advocacy groups should work together to co-develop: (1) patient-centric governance for gene therapy in Africa, (2) public engagement and education materials, and (3) decision making toolkits for trial participants. It is also critical to establish harmonised ethical and regulatory frameworks for gene therapy in Africa, and for global health agencies to accelerate access to basic care for SCD in Africa, while simultaneously strengthening capacity for gene therapy.

## Introduction

Somatic gene therapies have the potential to cure inherited disorders like cystic fibrosis, haemophilia, and sickle cell disease (SCD), and would likely be considered one of the most exciting aspects of genetic medicine in Africa. The first human somatic gene therapy trial took place in 1990 and involved two young girls with severe combined immune deficiency (SCID) a rare, inherited immune-system disorder [1, 2]. The girls, Ashanthi De Silva and Cynthia Cutshall, were 4 and 9 years old, respectively [1, 3]. Although Cynthia’s cells accepted the new genes to a small extent (about 1%), her immune response improved. Three decades later, both girls are doing well, they continue to receive conventional therapy for SCID, and have reportedly experienced a significant improvement in their quality of life [4]. However, it is important to also acknowledge that there have been some negative outcomes of somatic gene therapy trials. For example, in 1999, Jesse Gelsinger, an 18-year-old boy with a rare metabolic disorder experienced serious complications, including blood clotting and multiple organ failure, after participating in a gene therapy trial [5]. Jesse passed away within four days of the trial, and his death was the first to be directly attributed to gene therapy. Jesse’s father later filed a negligence lawsuit against the university and scientists responsible for the trial [6]. The stories of Ashanthi, Cynthia, and Jesse may evoke a range of emotions and reactions among patients considering gene therapy and/or their caregivers, but as research on gene therapy evolves, lessons may be learned from these cases.

Somatic gene therapy is gaining attention in Africa as a promising curative approach for sickle cell disease (SCD). This is for several reasons. Firstly, it holds the promise of offering a “new life” to individuals living with chronic health conditions that currently have no cure. Secondly, gene therapy may provide a cost-effective alternative to lifelong clinical interventions for certain diseases. A notable example is in the treatment of SCD, a monogenic blood disorder that typically manifests in early childhood and characterised by chronic anaemia, acute painful episodes, and potentially life-threatening complications, including stroke, as well as damage to vital organs such as the spleen, brain, kidneys, and lungs.

Each year, approximately 515,000 babies worldwide are born with SCD and the majority of these births, over 75%, occur in Africa [7]. Without appropriate treatment, about 30% of children with untreated SCD in Africa will not survive beyond the age of five [8, 9]. Hydroxyurea, an FDA-approved pharmacological treatment for SCD since 1998 [10], is available in few African countries but comes at a cost of 16.50–54.60 USD for a monthly course, rendering it unaffordable and inaccessible for many patients in Africa [11, 12]. Furthermore, its acceptance amongst patients and caregivers has been challenging primarily due to negative or uninformed perceptions of the drug [12, 13] and concerns about it’s potential side effects on fertility [14–16]. The development of new drugs for SCD has been slow, and the only approved curative option in Africa is hematopoietic stem cell transplantation (HSCT). The implementation of HSCT in many African countries is however beset with significant challenges, including but not limited to delayed immune reconstitution, heightened susceptibility to infection, paucity of matched-sibling donors and limited expertise and infrastructure for peri-transplant care [17]. These factors have limited the widespread adoption of HSCT, motivating the search for alternative curative approaches, most notably, gene therapy.

The first successful gene therapy trial for SCD was reported in 2017, marking a significant milestone in the development of advanced therapies for SCD [18]. Since then, there has been remarkable advancements in science and innovation for gene therapies for SCD [19], particularly gene addition and gene editing. Gene addition strategies target the mutated beta-globin gene responsible for SCD and enhance the production of non-sickled adult haemoglobin or introduce a gene with anti-sickling properties [20]. This approach holds great promise for improving the symptoms and complications associated with SCD. In parallel, somatic gene editing techniques leverage on the CRISPR-CAS 9 technology to enable precise modification of the genetic material to correct the underlying genetic mutation responsible for SCD.

At the 2023 *International Summit on Human Genome Editing* in London, Victoria Grey shared her personal experience of living with SCD and how CRISPR gene therapy has transformed her outlook for the future. She stated that *“At one point in my life, I stopped planning for the future because I felt I didn’t have one. Now, I can dream again without limitation.”* [21]. Similar sentiments are shared by Jimi Olaghere, who in 2020 participated in an SCD gene editing trial in the USA [22]. Their testimonies add a profoundly human dimension to the academic discourse on somatic gene therapy for SCD, and highlights the transformative impact that gene therapy can have on individuals living with SCD. Amidst these inspiring narratives, it is imperative to acknowledge that gene therapy for SCD is not devoid of ethical and social challenges. This paper highlights a range of potential ethical, legal, and social dilemmas that extend beyond affordability.

## Current perspectives on the ethics of somatic gene therapy in Africa

In Africa, the past five years have witnessed promising developments for somatic gene therapy for SCD, HIV/AIDS and haemophilia. In 2019 for example, some research funders and pharmaceutical companies announced a possible partnership to advance gene therapy research for SCD and HIV in Africa (Cohen and Kaiser 2019). Additionally, a number of African researchers [19, 23] and persons living with SCD [24] have expressed enthusiasm for gene therapy as a curative option for SCD. Despite these encouraging developments, there appears to be a dearth of discussions pertaining to the potential ethical, legal and social issues associated with gene therapy trials in Africa. To date, much of the discourse primarily revolves around the prohibitive cost of gene therapies and the regulatory void for gene therapy trials in many African countries [25, 26].

### Cost of gene therapy may make it a technology for the wealthy and affluent of society

The estimated cost of gene therapy for SCD per patient in Africa is unknown and discussions on it’s affordability have relied predominantly on estimates from high income countries, where the cost of gene therapy for monogenetic conditions currently exceeds 1.5 million USD per patient [27]. This exorbitant cost raises concerns about affordability for SCD patients in Africa, especially given that many African countries do not have national health insurance programs or universal health coverage [28] and rely heavily on out-of-pocket payments and/or external donor funding for healthcare [29]. Additionally, most families/patients, struggle to cover health expenses associated with basic SCD care, let alone the cost for managing SCD related complications [30]. In Nigeria, for example, basic SCD care costs approximately 300 USD per month [31, 32]; while in the Republic of Congo it amounts to about 110 USD [33]. Compared to the estimated cost of gene therapy, only the wealthy would be able to afford gene therapy, thereby creating inequities in access. While some may argue that gene therapy for SCD may eventually prove cost-effective compared to the lifelong expenses associated with conventional SCD management, estimates in South Africa suggest that gene therapy for HIV would be unaffordable to many patients and will exceed the lifetime costs of accessing antiretroviral therapy [34, 35]. A similar cost-effective analysis for SCD in Africa is needed but would likely yield a comparable conclusion, suggesting the need to identify strategies for reducing the cost of gene therapy.

The Global Gene Therapy Initiative (GGTI) has proposed the concept place-of-care bio-manufacturing as a means to reduce cost and improve access [36]. In Africa, this approach would be viable only if regional and global bodies such as the African Union and the World Health Organisation, support technology transfer hubs that could produce gene therapy products for the continent. This could follow the model used for mRNA COVID-19 vaccines platforms [37]. However, it is vital to recognise that the cost of gene therapy encompasses not only the factory price of the products but also associated clinical procedures such as chemotherapy, bone transplantation, and patient monitoring, some of which are prohibitively expensive or unavailable in many African countries. Therefore, a concerted global effort is needed to explore avenues for reducing the cost of gene therapy. This may involve measures such as regulating the global price of gene therapy products, fostering public-private partnerships for gene therapy product development and trials in Africa, and strengthening capacity for bone marrow transplantation, chemotherapy, and patient monitoring for advanced therapies.

### Dearth of legal and regulatory guidelines for gene therapy in Africa

Many African countries do not yet have national guidelines that specifically address human gene therapy trials. This presents a challenge in ensuring proper ethical and regulatory oversight for somatic gene therapy trials. In South Africa, where some regulatory mechanisms are in place, they are reportedly contradictory and ambiguous, and the involvement of multiple national departments may further complicate the process of obtaining ethics and regulatory approvals [38, 39]. This gap presents a unique opportunity to establish harmonise and comprehensive ethics and regulatory frameworks for somatic gene therapy trials for the African continent. The harmonised frameworks should align with international regulations while considering the African research and clinical landscape, including regulatory mechanisms for manufacturing or approving gene therapy products in Africa and access to holistic healthcare for SCD. Developing such comprehensive guidelines would require a coordinated effort involving African national medecine regulatory authorities, health research institutions, research ethics committees, pharmaceutical companies, patient support/advocacy  groups and international health agencies such as the WHO.

## Reflections on the ethical and social dilemmas of SCD gene therapy in Africa

While issues of equitable access and research regulation are undeniably important, there is a risk that by only focussing on access and affordability, global health research stakeholders run the risks of leaving researchers, patients, regulators, and policymakers with limited information on other possible ethical, legal and social issues that may arise during the design and implementation of gene therapy trials in Africa, including how to ensure that somatic gene therapy is conducted in a responsible and ethically sound manner. This paper aims to address this gap by shedding light on other important and potential ethical, legal, and social challenges of gene therapy trials for SCD in Africa (Table 1).

**Table 1:** Summary of ethical and social considerations for somatic gene therapy in Africa.

Ethical Dilemma	Mechanisms for addressing ethical dilemmas	Examples of responsible stakeholders
Informed Consent	• Patient education. • Co-creation of consent documents and educational materials with patient support groups. • Structure informed consent as a multi-stage process that begins with education, before selection of participants and evaluation of knowledge.	• Patient support/advocacy groups • Research funders • Researchers • Research ethics committees
Therapeutic mis-estimation, and optimistic bias	• Public engagement on SCD gene therapy • Honest and clear communication on risks and benefits • Training of journalist and science communicators on non-sensational media coverage of SCD gene therapy trials	• Patient support/advocacy groups • Research funders • Researchers
Decisional conflicts: Mature minors and young adults and their parents/caregivers,	• Use tools such as the MacArthur Competence Assessment Tool for Clinical Research to access decision making ability for gene therapy. • Involve patient support groups and counsellors to identify culturally appropriate strategies for navigating conflict. • Provide emotional and psychosocial support to both parents and adolescents/young adults to help them cope with the challenges that come with decisional conflict.	• Researchers • Patient support groups
Standards of care for SCD gene therapy trials in Africa.	• Multistakeholder engagement to ensure improved access to basic conventional therapies for SCD. • Determine appropriate standards of care for SCD Gene therapy. • Commitment to long-term follow-up of trial participants by study teams	• National public health agencies • Pharmaceutical companies • National governments/Ministries of Health
Risks/Benefit analysis	• Transparency on known and anticipated risks • Public and patient education on current uncertainties and risks of gene therapy	• Researchers • Pharmaceutical companies • Funders • National health regulatory authorities
Selection of participants, withdrawal and discontinuation of trial	• Define exclusion and inclusion criteria. • Consider inclusion of children/minors in gene therapy trials • Trial team should list clinical and psychosocial support available to participants following withdrawal or discontinuation of study. • Researchers should discuss access and use of patient data in the case of withdrawal from the study	• Researchers • Research ethics committees • National health regulatory authorities
Bounded Justice: Inequities in global access to gene therapy	• Develop governance and regulatory mechanisms that will enable African countries compete equitably in the global gene therapy enterprise. • Strengthen critical areas for gene therapy such as genetic medicine and clinical trial infrastructure. • Researchers and funders should ensure that SCD gene therapy trials adhere to the highest ethical standards, including equitable access to participation and care	• Global health agencies (e.g., WHO) • Research funders • National public health agencies/ research councils • Patient advocacy groups • National drug regulatory authorities • African research institutions • Pharmaceutical companies

### Transparency in communicating risks and adverse events of gene therapy

Somatic gene therapy for SCD carries potential risks, such as gene silencing, gene toxicity, phototoxicity and inadvertent germline transmission of DNA [40, 41]. Additionally, some studies reported a possible risk of developing leukaemia and myelodysplastic syndrome [42, 43]. Emerging evidence hints at the possibility of off-target editing with unforeseen long-term consequences [44]. While efforts are underway to minimise these risks, patients and their caregivers, research ethics committees and research regulators would need clear and honest communication about the possibility of these different risks, even if they are transient. This includes detailing how risks will be monitored and managed by the trial team, and how they compare to the risks associated with existing standards of care and other advanced therapies for SCD.

Given the rapidly evolving nature of SCD gene therapy and the unpredictable clinical course of SCD, it is also important to acknowledge the current lack of comprehensive information on the long-term benefits of gene therapy for SCD. Such open and honest communication is essential for informed decision-making by patients and their caregivers, as well as in fostering trust between potential participants and researchers. Furthermore, understanding public perceptions of these risks is crucial as it can facilitate risk communication during informed consent and community engagement and public education on gene therapy for SCD.

### Therapeutic mis-estimation and uncertainties about gene therapy

The possibility of a life free from the pain crises and complications associated with SCD, can create high expectations regarding the potential benefits of gene therapy [24]. As a consequence, there is a possibility that patients and their caregivers may perceive the risks associated with gene therapy as minimal compared to the long-term physical and psychological discomfort caused by SCD. This phenomenon, known as therapeutic misestimation, can lead to an underestimation of the actual risks of gene therapy and an unrealistic conviction of experiencing only the benefits (therapeutic optimism).

Several factors may contribute to therapeutic misestimation and optimism, such as religious beliefs, the hope for a miraculous cure and sensational or hyped media coverage of successful gene therapy trials. Consequently, some patients and their caregivers may consent for gene therapy without fully considering the potential risks and complications. In light of these, it is imperative for researchers, healthcare providers, and the media to communicate the experimental nature of gene therapy and the potential risks accurately, and without exaggeration, so as to enable potential participants to make decisions based on a realistic understanding of the procedure and its potential outcomes. Also, reserachers should work closely with genetic counsellors and patient advocacy groups to develop mechanisms and platforms for providing support sharing experiences, and facilitating peer-to-peer discussions, which can help individuals gain a more balanced perspective on gene therapy as well as enable informed decision making for participation in gene therapy trials.

### Criteria for selection of participants, withdrawal from study and discontinuation of trial

It is likely that gene therapy trials  for SCD will become available in Africa within the next decade. Consequently, it is essential to establish clear criteria (e.g., disease severity, comorbidities, age) for selecting patient-participants, procedures or withdrawal from the trial and conditions for discontinuation of the trial. In the United States, current protocols for SCD gene therapy exclude children based on the low SCD childhood mortality rates in the region [45]. However, this exclusion criteria may be debatable in sub-Saharan Africa, where many children living with SCD do not reach their fifth birthday due to limited access to comprehensive care [8, 46] and may benefit more from gene therapy. The worsening of SCD with age further complicates the decision-making process, especially as there is limited information on health-related quality of life across the lifespan of individuals living with SCD in Africa. If the goal of SCD gene therapy is to improve overall health-related quality of life, there may a valid case for including children in SCD gene therapy trials in Africa. This would provide data on how gene therapy compares to existing standards of care for SCD in African countries or other advanced therapies. It would also enable a comparison of outcomes, and help patients and their families make more informed decisions regarding their preferred intervention.

While it is standard practice that participants can withdraw from a clinical trial at any time, this can be tricky for gene therapy trials once the actual procedure has commenced. Possibility of withdrawal only seems possible in terms of withdrawing their data from the study. Addressing the challenge of withdrawal, may require tailoring consent as a dynamic process, where in the initial stages, the focus is more on improving patients and caregiver’s knowledge of gene therapy trials, such that before the intervention is administered, the patient would have had enough time to reflect on, and make an informed decision about their participation in the trial.

As with study withdrawal, discontinuation, or premature termination of a gene therapy trial, be it for reasons related to serious adverse events, access to participants or logistical challenges can lead to uncertainties for trial participants. Also, considering that long term follow up is required to ascertain risk post-trial and detect potential delayed side effects, investigators will need to inform participants about the alternative care and psychosocial support that would be made available to trial participants.

### Informed consent: comprehension and balancing hope and uncertainty

While gene therapy has been presented as a potential one-time cure for SCD, there are risks related to gene silencing, gene toxicity, phototoxicity, uncertainties (even if transient) around germline transmission of DNA, and viral shedding. It is important to acknowledge the current lack of comprehensive information on long-term benefits, including clarity on whether gene therapy will prevent occurrences of chronic organ dysfunction and failure in SCD [47]. It is important to ensure that the optimism of scientists and clinicians do not influence the final decision of patients and that potential trial participants are clear about the potential benefits, risks and uncertainties of gene therapy. To this effect, informed consent for SCD gene therapy should be designed such that the first stage involves education of patients (or parents/guardians) about the procedures, potential benefits and risks, possibilities of withdrawal and procedures for long term monitoring, followed by assessment of literacy and comprehension. We recommend that the planning stages of gene therapy trials/research for SCD in Africa should prioritise the development and testing of consent documents and educational materials with patient support groups, as this could enable patient participants and their caregivers to have fore knowledge of gene therapy, its limitations and risks, even before they are approached to be part of a trial. The co-creation of these documents and materials with patient support groups will not only help improve understanding and decision making, but also foster discussions amongst various stakeholders on the ethical and social aspects of somatic gene therapy for SCD.

### Decisional conflict based on varying concerns and priorities for parents and their children, especially mature minors, and young adults

Consent for gene therapy involving minors, especially adolescents can present complex moral dilemma often involving conflicting decisions between parents and their children regarding trial participation. For example, a mature minor living with SCD may be more optimistic about the potential of a cure even if their parents are reluctant to consent. In designing gene therapy trials, it is important to appreciate that both perspectives-those of the adolescent and the parents-are valid, and stem from distinct concerns. Therefore, when developing educational and patient engagement interventions it is imperative to consider these divergent viewpoints.

Minimising decisional conflict in consent between parents and mature minors would require that mechanisms to assess the ability of mature minors and their parents to understand relevant information, appreciate the potential risks and benefits and to make an informed decision. Providing psychosocial support to both parents and adolescents/young adults to help them cope with the challenges that come with decisional conflict will also be key. The MacArthur Competence Assessment Tool for Clinical Research [48], is an example of a literacy and decision making assessment tool that could be used to access the ability of mature minors and young adults to provide informed consent for SCD gene therapy trials. Bearing in mind that the cultural context in Africa plays a major role in decision making within families, engagements with SCD patient support groups can help identify culturally and appropriate strategies to navigate decisional conflict in gene therapy trials in Africa. Open and inclusive discussions with adolescents/young adults their caregivers, health care providers ad patient support groups could also help clarify expectations and clarify concerns ultimately leading to decisions that are in the best interest of the minor or young adult.

### Appropriate standards of care for SCD gene therapy trials in Africa

Patients who participate in gene therapy trials will require complex care before, during, and after the trial, it is necessary to establish and communicate to ethics committees and potential participants, the standards of care that was considered in the design of the trial and what will be available to trial participants at the end of the study. Comprehensive SCD healthcare is not available in many African countries and Hydroxyurea, which has been described as the go to drug for improving the quality of life of persons living with SCD [49], is not yet accessible to many patients in Africa [12, 50, 51]. Also, considering that long term follow up is required to ascertain risk and delayed adverse events, investigators will need to inform participants about the measures in place to ensure quality care, and in the case of discontinuation of the trial their commitment to ensuring that trial participants receive support and comprehensive care. SickleInAfrica [52], have proposed multi-level standards of care guidelines for SCD in Africa [53]. However, broad stakeholder engagement would be required to improve access to basic SCD medications and interventions as listed for example in the SickleInAfrica standards of care guidelines for SCD. Without wide access to basic care for SCD in Africa, SCD gene therapy trials in Africa risk being flagged as exploitative or extractive research.

### Bounded Justice: Inequities in global access to SCD gene therapy trials

It would be an injustice in global health if a continent that bears 75% of the global SCD burden is unable to access a cure for SCD when it becomes available or be able to access and participate in trials for a cure. Currently, there are no SCD gene therapy trials ongoing in Africa and there is an ongoing debate on whether limited research resources should be allocated to gene therapy, when access to basic lifesaving interventions such as prophylactic penicillin, hydroxyurea and folic acid is still a major challenge in many African countries [54]. Policy makers in many African countries may also contend that there are cost-effective treatments that could improve the quality of life of persons living with SCD. Nonetheless, the high upfront cost of gene therapy should not be reason for tardiness in promoting gene therapy trials for SCD Africa, considering that the lifetime cost (by age 50) of managing a patient living with SCD exceeds eight million USD (Leonard et al., 2020). This health economics argument can be persuasive, especially for those who can afford it. Alternatively, individuals living with SCD may advocate for access to gene therapy from a critical utilitarian perspective-that it will improve their quality of life. This is exemplified in the story of Jimi Olaghere who, in 2020, consented to an SCD gene editing trial in the USA [22]. Jimi explained how SCD infiltrated every aspect of his life including having to leave his parents in Nigeria to stay with relatives in the USA in order access better care, but how as he got older, his pain episodes were frequent, excruciating and he would sometimes wake up in intensive care feeling disappointed to still be alive [55]. A year, following his participation in the gene therapy trial (watched by his parents in Nigeria via a live stream), Jimi says he is now able to plan for decades in the future, unlike before. Many SCD patients in Africa and their caregivers are likely to share similar sentiments as Jimi and, therefore, advocate for researchers, funders and governments to prioritise gene therapy for SCD.

A major contributing factor to global inequities in access to gene therapy for SCD has to with broader national health system challenges in many African countries. This includes a dearth of infrastructural capacity for specialised clinical care. Currently, available gene therapies for SCD require chemotherapy and bone marrow transplant, yet these services are largely unavailable or not fully functional in many African countries [56, 57]. The consequence is that the wealthy will travel to high income countries to access gene therapy, leading to the unintended consequence of “genetic tourism”. Medical tourism, of which genetic tourism, is a subset also raises ethical and legal issues that will also need to be considered in discussions on the governance of gene therapy. It would be important to define, and be transparent, about how priorities for SCD gene therapy trials would be made in Africa. At the early stages priority areas should undoubtedly be on building ethics and regulatory and biosafety capacity, patient education and public engagement and clinical infrastructure to host SCD gene therapy trials. Thereafter, the focus can shift to finding affordable costing models, and by extension equitable access to SCD gene therapy.

Global and public health agencies together with pharmaceutical companies have a moral obligation to uphold the concept of non-abandonment and give equal value to the lives of persons living with this long term and incurable condition, by allowing them access to specialised care even if it would preclude allocation of scientific and public health resources in the most cost-effective way [58]. Similar arguments have been made for rare medical conditions [59] and orphan drugs for SCD [60]. Otherwise, we may witness a situation where the most vulnerable, would have travel to HICs to access gene therapy for SCD, exposing them to the unintended consequence of “genetic tourism”, including exploitation by commercial services. This exploitation make take the form of extravagant claims about the outcomes of gene therapy, commercial and commercial health services downplaying the risks involved and providing little clarity on follow up procedures, especially for medical tourists .

## Looking ahead: governance, capacity strengthening, Patient Education and public engagement for gene therapy in Africa

It is crucial to have open and transparent dialogues on the ethics of SCD gene therapy in Africa, establish governance mechanisms that are grounded in global health justice, local norms, and cultural values and strengthen capacity of gene therapy trials. Additionally, there will be a need for a comprehensive public engagement and education plan for gene therapy in Africa.

### Capacity strengthening genetic medicine and bio-manufacturing

Gene therapy will require haematopoietic stem cell transplant, chemotherapy and long term follow up of trial participants. Capacity for these services is largely missing in many African countries. On the ethics and regulatory arm, research ethics committees in Africa may lack the necessary expertise to provide scientific and ethical oversight for gene therapy research. As a result, there is a risk that important ethical issues may be overlooked or that approvals would be delayed. Furthermore, the absence of national regulations for gene therapy trials in many African countries may further complicate the ethical oversight process.

Engaging key stakeholders early in the process, especially in relation to manufacturing, licencing, regulatory approvals, and cost-effective importation of gene therapy products will be essential. Gene therapy initiatives for SCD may leverage on existing SCD clinical and genetic research initiatives in Africa. SickleInAfrica [52] for example, has set up an operational SCD registry in eight African countries and has experience engaging with patient support groups and in addressing ethical issues related to both SCD and genetics research in Africa [61–63]. Nigeria, Ghana, South Africa, and Tanzania, four of SickleInAfrica participating country sites also have centres and or registries for bone marrow transplant [56, 64] thereby making it easy to coordinate and support ethical gene therapy trials in Africa. SCD gene therapy.

### Governance: mapping the interest and responsibilities of different stakeholders

Stakeholders for SCD gene therapy trials in Africa include, but are not limited to, patients and their families/caregivers, patient support groups, healthcare workers, scientists, funders, ethics review committees, national drug regulatory bodies, scientific journals, mainstream media, and biotechnology/pharmaceutical companies. It is necessary to identify and map the respective roles, responsibilities, and interest (financial and otherwise) of these different stakeholders. This includes responsibilitie around the cost of trials; ensuring reasonable access to gene therapy; how data from gene therapy trials would be used; and how issues of gene tourism be addressed.

### Public engagement, patient education and counselling

Currently, there is little available information on public attitudes towards curative options for SCD. This leaves a knowledge gap on how to approach gene therapy trials for SCD in ways that are socially and culturally acceptable. Meaningful engagement of patient support and advocacy groups during the pre-clinical stages of gene therapy trials in Africa will be essential in improving decision-making, as well as fostering public trust in somatic gene therapy. Scientists and clinicians should be aware that if patients, and the public, have unmet expectations about gene therapy for SCD, they may end up disappointed and/or distrustful of the research enterprise. This will also be the case if scientists and the media fail to responsibly report on the results (positive and negative) of gene therapy trials. The accuracy, timeliness, and tone of media reports, as well as transparency in engaging with the media, are all critical, particularly in reporting serious adverse events. Initiatives such as SickleInAfrica have started exploring ways of engaging stakeholders in public health genetics [65] and this experience could be leveraged for SCD gene therapy research in Africa. Public engagement initiatives should see patient-participants as partners who have the agency to contribute to the implementation of SCD gene therapy trials. It will be important to empower patient support groups by co-developing educational materials on the science and ethics of gene therapy for SCD.

## Conclusion

The ELSIs for somatic gene therapy for SCD are complex but can be effectively addressed through a comprehensive approach involving various stakeholders and SCD initiatives. Using a variety of approaches including patient-centred governance, academic-industry partnerships, developing creative ways of enabling informed decision making for consent to participate in gene therapy trials, public engagement, and patient education. Establishing centres of excellence for SCD in Africa that can serve as hubs for SCD research, treatment, and capacity building, while supporting gene therapy trials would be a much more responsible and ethical approach to pursuing SCD gene therapy trails in a region that has a high SCD burden, yet limited access to basic clinical and psychosocial SCD care. The rapid development of COVID-19 vaccines has demonstrated that if there is sufficient will, global health actors can accelerate global equitable access to advanced health technology, such as gene therapy. We are of the opinion that the World Health Organisation, pharmaceutical companies, research funders and global health advocacy groups could do the same to support the implementation of gene therapy research and trials for SCD in Africa.
